# A sealant with a hemostatic mechanism independent of the blood coagulation function was effective in both elective and emergency surgery for thoracic aorta

**DOI:** 10.1007/s11748-023-01918-7

**Published:** 2023-03-14

**Authors:** Shigeki Morita, Hitoshi Yaku

**Affiliations:** 1 Department of Cardiovascular Surgery, National Hospital Organization Kyushu Medical Center, Fukuoka, Fukuoka, 810-8563 Japan; 2 Department of Cardiovascular Surgery, Kyoto Prefectural University of Medicine, Kyoto, Japan

**Keywords:** Surgical sealant, Thoracic aorta, Acute aortic syndrome, Vascular anastomosis, Blood coagulation system

## Abstract

**Objectives:**

Matsudaito is a unique surgical sealant with a powerful hemostatic effect that works independent of a patient’s blood coagulation function. Because of its mechanism, this sealant is expected to be particularly useful in patients with a poor blood coagulation function, such as in cases of acute aortic syndrome requiring emergency surgery. We, therefore, evaluated the hemostatic static effect of the sealant in both emergency and elective surgery of the thoracic aorta.

**Methods:**

We used data obtained from post-marketing surveillance of the sealant. Patients who underwent replacement of the thoracic aorta were enrolled. The hemostatic effect was evaluated as effective if a further hemostatic procedure was not performed after applying the sealant.

**Results:**

From 46 hospitals in Japan, a total of 542 patients (327 elective and 215 emergency cases) were enrolled. Hospital mortality was 4.0% and 11.6% in elective and emergency cases, respectively (*p* < 0.05). Among the 1039 anastomoses (609 elective and 430 emergency cases), effective hemostasis was confirmed in 436 (71.6%) elective and 259 (60.2%) emergency cases. The data from the clinical trial of the sealant showed a hemostatic rate of 44.4% in elective control cases without the sealant.

**Conclusion:**

Given that the hemostatic rate in emergency surgery with the sealant seemed to be better than that in elective surgery without the sealant (determined from the clinical trial), we concluded that the sealant was effective in both emergency and elective thoracic surgery of the aorta.

## Introduction

Effective hemostasis is key to the success of aortic surgery. Numerous surgical sealants had been developed for controlling bleeding [1–4]. However, currently available sealants have several limitations, such as risk of contamination with microbiological agents because of the biological nature of the source of the sealants [5], dependence on the coagulation function of the patients (which is not always intact) [6], toxic properties of the component of the sealant sometimes causing tissue injury [7], and non-elastic property of the cured sealant sometimes causing thinning of the aortic wall [8, 9].

**Fig. 1:**
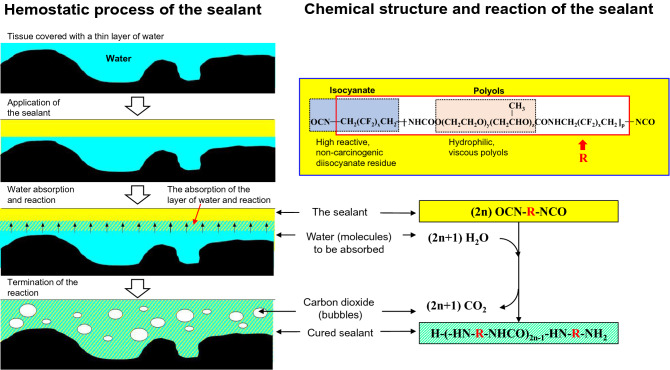
Hemostatic mechanism of the sealant

To resolve these issues, an elastometric surgical sealant (Matsudaito; Sanyo Chemical Industries Ltd., Kyoto, Japan; brand name “AQUABRID” in Europe and “Hydrofit” in Japan) was developed with a highly reactive copolymer of polyethylene glycol (PEG) and polypropylene glycol (PPG), in which both ends were capped with non-carcinogenic fluorinated hexamethylene diisocyanate [10]. The sealant is provided as a viscous liquid in a syringe. Once applied to the anastomosis, the sealant of the isocyanate group reacts with water molecules, which initiates the polymerization reaction (Fig. 1). Therefore, this sealant does not need any additional agents to initiate the reaction. After polymerization, the sealant exhibits an elastomeric property, following the pulsatile movement of the aorta. The sealant can be used under relatively harsh conditions, such as fully heparinized conditions before protamine sulfate administration and/or depleted coagulation factors or platelets, as it starts its reaction only with water and does not depend on the coagulation factor of the blood, unlike many other sealants. The sealant might also be effective in patients with acute aortic syndrome (AAS), whose blood coagulation function is impaired.

A clinical trial of the sealant was performed for the approval indicated by Pharmaceuticals and Medical Devices Agency of Japan (PMDA). The trial, however, was performed only for elective cases, and patients with AAS requiring emergency surgery were not included [11]. The sealant was approved for vascular anastomoses between vascular prosthesis and the thoracic aortae or their branched arteries. The regulation of The Ministry of Health Labor and Welfare of Japan (MHLW) requests that newly developed medical devices undergo post-marketing surveillance (PMS). This regulation was applied for the sealant, and the investigation was requested to be performed in accordance with Good Post-marketing Surveillance Practice (GPSP) Ministerial Ordinance [12].

The purpose of such PMS is to search further for any safety issues. To this end, the PMDA requested large-volume data (more than 500 patients) be gathered, with patients followed for 1 year. The data obtained from the PMS were real-world data and included both elective and emergency surgery of the thoracic aorta. We, therefore, capitalized on the fact that the data included AAS patients and hypothesized that the hemostatic function of the sealant would be comparably effective in emergency surgery for AAS because of its unique hemostatic mechanism. Our findings are reported herein.

## Patients and methods

The data were obtained from the PMS of the sealant, in which 46 hospitals in Japan participated. Consecutive patients, including those who underwent both elective and emergency surgery for AAS, were enrolled. The study was approved by the internal review board of each hospital. Informed consent was waived by the law because of the nature of the PMS.

### Surgical sealant

The sealant is a viscous liquid of diisocyanate-endcapped prepolymer. It is provided in aliquots of 2 g as a viscous liquid pre-filled in a syringe. The liquid PEG–PPG copolymer segment has a very high water-uptake characteristic, and the fluorinated isocyanate group reacts with water to form an elastic cured film after polymerization.

The major favorable feature of this sealant is its hydrophilic property, which allows its penetration into depressions on the tissue surface or needle holes while absorbing water molecules (Fig. 1). The seal is non-absorbable and remains in the body for long periods of time.

### Data gathering protocol of the surveillance

The surgeons in each hospital were asked to inform the research office when they decided to register a patient. A mandatory report was requested immediately after the patient was discharged and one year after the surgery. When death or major adverse events occurred, additional reports were requested.

### Evaluating the hemostatic effect

The surgeons were asked to use the sealant for anastomoses when hemostasis was required after completing the anastomosis. The timing of sealant application (before or after protamine administration) was left to the surgeon, but it had to be recorded on the report sheet. For each anastomosis, the hemostatic effect was evaluated and was recorded as “effective” if the bleeding stopped without any additional hemostatic procedure, such as the application of surgical stitches or other hemostatic agents. If additional hemostatic procedures were performed, the hemostasis of the sealant was evaluated as “not effective.”

### Statistical analysis

For categorical variables, a chi-squared test was used for the statistical comparison. For continuous variables, a Student ‘s *t*-test was used for the statistical comparison. A two-sided significance level of 5% and a two-sided confidence level of 95% were used to determine the significance of differences between the groups.

## Results

### Patients and background

Between June 12, 2014, and July 27, 2015, a total of 542 patients were enrolled. Of these, 327 underwent elective surgery, while 215 underwent emergency surgery due to AAS. The demographics and perioperative information of these patients are summarized in Table 1. There were no marked differences between the groups in demographics except for the male/female ratio (more men in elective surgery) and hypertension (more hypertensive patients in the emergency surgery group).

**Table 1:** Patients’ background and characteristics

Items	Category	N^*1^			*p*-value^*2^
		Total *N* = 542	Elective *N* = 327	Emergency *N* = 215	
Gender	Male	354 (65.3%)	235 (71.9%)	119 (55.3%)	< 0.001
	Female	188 (34.7%)	92 (28.1%)	96 (44.7%)	
Comorbidity	Hypertension	194 (35.8%)	102 (31.2%)	92 (42.8%)	0.006
	Hyperlipidemia	44 (8.1%)	30 (9.2%)	14 (6.5%)	0.267
	Diabetes	32 (5.9%)	19 (5.8%)	13 (6.0%)	0.909
	Cerebrovascular disease	4 (0.7%)	3 (0.9%)	1 (0.5%)	0.547
Age	Average age <years old>	66.6 (SD.13.0)	66.2 (SD.13.0)	67.3 (SD.12.9)	0.314
	Over 80 years old	88 (16.2%)	48 (14.7%)	40 (18.6%)	0.236
Replaced aorta	Aortic root	2 (0.4%)	2 (0.6%)	0 (0.0%)	0.251
	Root and Ascending aorta	14 (2.6%)	13 (4.0%)	1 (0.5%)	0.012
	Ascending aorta^*3^	154 (28.4%)	68 (20.8%)	86 (40.0%)	< 0.001
	Root and partial aortic arch	1 (0.2%)	1 (0.3%)	0 (0.0%)	0.417
	Ascending aorta and partial aortic arch	76 (14.0%)	34 (10.4%)	42 (19.5%)	0.003
	Partial aortic arch and Descending aorta	2 (0.4%)	2 (0.6%)	0 (0.0%)	0.251
	Root and Total aortic arch	3 (0.6%)	3 (0.9%)	0 (0.0%)	0.159
	Total aortic arch^*4^	228 (42.1%)	149 (45.6%)	79 (36.7%)	0.042
	Total aortic arch and Descending aorta	3 (0.6%)	1 (0.3%)	2 (0.9%)	0.338
	Descending aorta	32 (5.9%)	28 (8.6%)	4 (1.9%)	0.001
	Thoraco-abdominal aorta	27 (5.0%)	26 (8.0%)	1 (0.5%)	< 0.001
Concomitant procedure	Performed	171 (31.5%)	136 (41.6%)	35 (16.3%)	< 0.001

*^1 ^*N*: number of patients

*^2^All *p* values are provided with χ2 test except for "Average age" given with* t*-test

*^3 ^There were 5 redo patients (pseudoaneurysm: 4, graft infection: 1)

*^4 ^There was one redo patient (graft infection: 1)

The most frequently performed procedure in the elective group was total arch replacement (149 patients, 45.6%), whereas that in the emergency group was ascending aorta replacement (79 patients, 36.7%).

### Hemostatic effectiveness

**Fig. 2:**
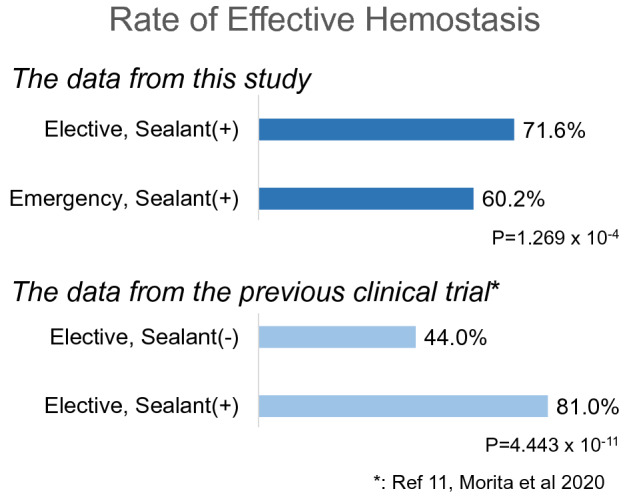
Top: Overall hemostatic effectiveness of the sealant from the current PMS. Bottom: Hemostatic effectiveness of the sealant from the previous clinical trial (Morita [11])

The sites of anastomoses where the sealant was applied are shown in Table 2. There were 609 anastomoses in the elective group and 430 in the emergency group. Hemostatic effectiveness is shown in Table 3A. When the sealant was applied before protamine administration, effective hemostasis was obtained in 69.0% and 52.3% of cases in the elective and emergency groups, respectively; these percentages rose to 73.4% and 63.7%, respectively, when the sealant was applied to the anastomoses after the administration of protamine. We further looked into the relation between the type of vessels (large aorta versus branched arteries) and hemostatic effectiveness. The results are shown in Table 3B. The hemostatic effectiveness was better when the sealant was applied to the branched artery anastomoses (72.2%) than that of the large vessels (64.9%). The differences, however, were lost when the analysis was applied to subgroups such as emergency surgery and elective surgery groups. The overall hemostatic effectiveness was 71.6% and 60.2% in the elective and emergency groups, respectively (Fig. 2).

**Table 2:** Anastomotic sites where the sealant was applied

Category	n^*1^	Details		n^*1^			*p*-value (χ2 test)
				Total *n *= 1,039	Elective *n *= 609	Emergency *n *= 430	
Anastomoses of thoracic aorta replacement	993 (95.6%)	Aortic root		20 (1.9%)	19 (3.1%)	1 (0.2%)	0.001
		Ascending aorta	Proximal	252 (24.3%)	123 (20.2%)	129 (30.0%)	< 0.001
			Graft-to-graft	89 (8.6%)	52 (8.5%)	37 (8.6%)	0.970
			Distal	124 (11.9%)	57 (9.4%)	67(15.6%)	0.002
		Aortic branch	BCA^*2^	83 (8.0%)	43 (7.1%)	40 (9.3%)	0.189
			LCCA^*2^	64 (6.2%)	37 (6.1%)	27 (6.3%)	0.893
			LSCA^*2^	58 (5.6%)	39 (6.4%)	19 (4.4%)	0.170
			LSCA (graft-to-graft)	7 (0.7%)	3 (0.5%)	4 (0.9%)	0.396
		Aortic arch	Hemiarch (distal)	11 (1.1%)	1 (0.2%)	10 (2.3%)	0.001
			BCA-LCCA	8 (0.8%)	3 (0.5%)	5 (1.2%)	0.224
			LCCA-LSCA	10 (1.0%)	6 (1.0%)	4 (0.9%)	0.929
			Aortic arch-graft (island reconstruction)	1 (0.1%)	0 (0.0%)	1 (0.2%)	0.234
			Graft-to-graft (distal)	95 (9.1%)	61 (10.0%)	34 (7.9%)	0.245
			Distal	81 (7.8%)	52 (8.5%)	29 (6.7%)	0.288
		Descending aorta	Proximal	49 (4.7%)	47 (7.7%)	2 (0.5%)	<0.001
			Graft-to-graft	9 (0.9%)	6 (1.0%)	3 (0.7%)	0.622
			Distal	21 (2.0%)	19 (3.1%)	2 (0.5%)	0.003
			Intercostal artery	8 (0.8%)	8 (1.3%)	0 (0.0%)	0.017
			Intercostal artery (graft-to-graft)	1 (0.1%)	1 (0.2%)	0 (0.0%)	0.401
		Others	Total debranch	1 (0.1%)	1 (0.2%)	0 (0.0%)	0.401
			The 3rd branch in neck (graft) - left axillary bypass (graft)	1 (0.1%)	1 (0.2%)	0 (0.0%)	0.401
Other sites	46 (4.4%)	Coronary artery reconstruction site or bypass site		6 (0.6%)	5 (0.8%)	1 (0.2%)	0.218
		Abdominal aortic replacement site		5 (0.5%)	5 (0.8%)	0 (0.0%)	0.060
		Abdominal bifurcation artery replacement site		5 (0.5%)	5 (0.8%)	0 (0.0%)	0.060
		Heart surface		4 (0.4%)	1 (0.2%)	3 (0.7%)	0.171
		Native blood vessel surface		2 (0.2%)	2 (0.3%)	0 (0.0%)	0.234
		Sending or removing blood site and air bleeding site		24 (2.3%)	12 (2.0%)	12 (2.8%)	0.386

*^1 ^*n*: number of the sites where the sealant was applied

*^2 ^*BCA* brachiocephalic artery, *LCCA* left common carotid artery, *LSCA* left subclavian artery

**Table 3:** Hemostatic effectiveness for each anastomosis

A. Hemostatic effectiveness compared between elective and emergency surgeries								
*(a) Application timing; Before protamine sulfateadministration*					Total *n*=385^*1^	Elective *n*= 255	Emergency *n*=130	*p*-value (χ2 test)
Effective					244 (63.4%)	176 (69.0%)	68 (52.3%)	0.001
Not effective					141 (36.6%)	79 (31.0%)	62 (47.7%)	
*(b) Application timing; After protamine sulfateadministration*					Total *n*=654^*1^	Elective *n*= 354	Emergency *n*=300	*p*-value (χ2 test)
Effective					451 (69.0%)	260 (73.4%)	191 (63.7%)	0.007
Not effective					203 (31.0%)	94 (26.6%)	109 (36.3%)	
*(c) Overall results*					Total *n*=1039^*1^	Elective *n*= 609	Emergency *n*=430	*p*-value (χ2 test)
Effective					695 (66.9%)	436 (71.6%)	259 (60.2%)	<0.001
Not effective					344 (33.1%)	173 (28.4%)	171 (39.8%)	
^*1^ The sealant was used before neutralizing heparin in 37.1% of anastomoses (385/1039) and after neutralizing heparin in 62.9% of anastomoses (654/1039).								

B. Hemostatic effectiveness compared between the large vessel and branched vessel anastomoses								
*(a)Electve*					Total *n*=579^*2^	Large vessel *n*= 446	Branch *n*=133	*p*-value (χ2 test)
Effective					416 (71.8%)	316 (70.9%)	100 (75.2%)	0.329
Not effective					163 (28.2%)	130 (29.1%)	33 (24.8%)	
*(b)Emergency*					Total *n*=414^*2^	Large vessel *n*= 324	Branch *n*=90	*p*-value (χ2 test)
Effective					245 (59.2%)	184 (56.8%)	61 (67.8%)	0.061
Not effective					169 (40.8%)	140 (43.2%)	29 (32.2%)	
*(c)Overall results*					Total *n*=993^*2^	Large vessel *n*= 770	Branch *n*=223	*p*-value (χ2 test)
Effective					661 (66.6%)	500 (64.9%)	161 (72.2%)	0.043
Not effective					332 (33.4%)	270 (35.1%)	62 (27.8%)	
^*2^ The sealant was used in elective surgery in 58.3% of anastomoses (579/993) and in emergency surgery in 41.7% of anastomoses (414/993).

### Deaths and adverse events

All deaths are summarized in Table 4. A total of 38 patients died before discharge, including 13 (4.0%) in the elective group and 25 (11.6%) in the emergency group (*p* = 0.001). After discharge, 16 patients (4.9%) in the elective group and 12 (5.6%) in the emergency group died (*p* = 0.723). Twenty-nine patients were lost after discharge. To compare the death rate before discharge in each category (emergency vs. elective, true aneurysm vs. aortic dissection), annual data from the Japanese Association for Thoracic Surgery [13] are shown at the bottom of Table 4. The results were comparable in all categories.

**Table 4:** Death after surgery

		Elective			Emergency		
		Unruptured aortic aneurysm	Chronic aortic dissection	Sub-total	Ruptured aortic aneurysm	Acute aortic dissection	Sub-total
Data of this study^*1^							
	N^*2^	269	58	327	22	193	215
Mortality	30 Days	3 (1.1%)	1 (1.7%)	4 (1.2%)	3 (13.6%)	12 (6.2%)	15 (7.0%)
	Hospital	9 (3.3%)	4 (6.9%)	13 (4.0%)^*^^3^	7 (31.8%)	18 (9.3%)	25 (11.6%)^*3^
	After discharge	15 (5.6%)	1 (1.7%)	16 (4.9%)^*4^	1 (4.5%)	11 (5.7%)	12 (5.6%)^*4^
Data from The Japanese Association for Thoracic Surgery (2017) [Ref.13]^*1^							
	N^*2^	7781	2418	10199	448	6153	6601
Mortality	30 Days	242 (3.1%)	81 (3.3%)	323 (3.2%)	80 (18.3%)	564 (9.2%)	644 (9.8%)
	Hospital	365 (4.7%)	118 (4.9%)	483 (4.7%)	104 (23.0%)	681 (11.1%)	785 (11.9%)
	After discharge	N/A	N/A	N/A	N/A	N/A	N/A

*^1^Both studies do not include TEVAR except for open stent graft and debranching TEVAR

*^2^*N*: number of patients

*^3^χ^2^ test between sub-totals of elective and emergency: *p* = 0.001

*^4^χ^2^ test between sub-totals of elective and emergency: *p* = 0.723

The adverse events observed after surgery are shown in Table 5. Although the sealant persists around the anastomosis for long period of time, there was no particular increase in the incidence of inflammation or infection. The incidence of re-sternotomy for hemostasis was 2.2%.

**Table 5:** Adverse events

Adverse events			N^*1^			*p*-value (χ2 test)
			Total *N* = 542	Elective *N *= 327	Emergency *N *= 215	
Infection, inflammation	Pleural effusion	Total	115 (21.1%)^*2^	60 (18.2%)^*2^	55 (25.5%)	0.053
		Before discharge	110 (20.2%)^*2^	56 (17.0%)^*2^	54 (25.0%)	0.029
		After discharge	6 (1.1%)^*2^	5 (1.5%)^*2^	1 (0.5%)	0.410
	Pericardial effusion	Total	44 (8.1%)^*3^	22 (6.7%)^*3^	22 (10.2%)	0.151
		Before discharge	41 (7.5%)^*3^	19 (5.8%)^*3^	22 (10.2%)	0.068
		After discharge	4 (0.7%)^*3^	4 (1.2%)^*3^	0 (0.0%)	0.155
	Pyrexia higher than 38°C	Total	37 (6.8%)	15 (4.6%)	22 (10.2%)	0.014
		Before discharge	37 (6.8%)	15 (4.6%)	22 (10.2%)	0.014
		After discharge	0 (0.0%)	0 (0.0%)	0 (0.0%)	1.000
	Superficial incisional infection	Total	6 (1.1%)	3 (0.9%)	3 (1.4%)	0.686
		Before discharge	5 (0.9%)	3 (0.9%)	2 (0.9%)	1.000
		After discharge	1 (0.2%)	0 (0.0%)	1 (0.5%)	0.396
	Mediastinitis	Total	14 (2.6%)	8 (2.4%)	6 (2.8%)	0.790
		Before discharge	12 (2.2%)	7 (2.1%)	5 (2.3%)	1.000
		After discharge	2 (0.4%)	1 (0.3%)	1 (0.5%)	1.000
	Anastomosis site infection	Total	3 (0.6%)	0 (0.0%)	3 (1.4%)	0.062
		Before discharge	2 (0.4%)	0 (0.0%)	2 (0.9%)	0.158
		After discharge	1 (0.2%)	0 (0.0%)	1 (0.5%)	0.398
	Pneumonia	Total	27 (5.0%)^*4^	12 (3.7%)	15 (6.9%)^*4^	0.106
		Before discharge	24 (4.4%)^*4^	11 (3.4%)	13 (6.0%)^*4^	0.199
		After discharge	4 (0.7%)^*4^	1 (0.3%)	3 (1.4%)^*4^	0.306
Neurological	Paraparesis	Total	9 (1.7%)	6 (1.8%)	3 (1.4%)	1.000
		Before discharge	9 (1.7%)	6 (1.8%)	3 (1.4%)	1.000
		After discharge	0 (0.0%)	0 (0.0%)	0 (0.0%)	1.000
	Stroke	Total	35 (6.4%)	15 (4.6%)	20 (9.3%)	0.033
		Before discharge	34 (6.2%)	15 (4.6%)	19 (8.8%)	0.069
		After discharge	1 (0.2%)	0 (0.0%)	1 (0.5%)	0.396
Others	Atrial fibrillation	Total	15 (2.8%)	12 (3.7%)	3 (1.4%)	0.179
		Before discharge	15 (2.8%)	12 (3.7%)	3 (1.4%)	0.179
		After discharge	0 (0.0%)	0 (0.0%)	0 (0.0%)	1.000
	Bleeding requiring re-sternotomy	Total	12 (2.2%)	7 (2.1%)	5 (2.3%)	1.000
		Before discharge	12 (2.2%)	7 (2.1%)	5 (2.3%)	1.000
		After discharge	0 (0.0%)	0 (0.0%)	0 (0.0%)	1.000
	Respiratory failure requiring tracheostomy	Total	7 (1.3%)	2 (0.6%)	5 (2.3%)	0.121
		Before discharge	7 (1.3%)	2 (0.6%)	5 (2.3%)	0.121
		After discharge	0 (0.0%)	0 (0.0%)	0 (0.0%)	1.000
	Renal failure requiring dialysis	Total	9 (1.7%)	4 (1.2%)	5 (2.3%)	0.494
		Before discharge	8 (1.5%)	3 (0.9%)	5 (2.3%)	0.275
		After discharge	1 (0.2%)	1 (0.3%)	0 (0.0%)	1.000

*^1^*N* number of patients

*^2^One patient developed pleural effusion once before and after discharge

*^3^One patient developed pericardial effusion once before and after discharge

*^4^One patient developed pneumonia once before and after discharge

## Discussion

Utilizing the data from the PMS of the recently approved surgical sealant, we showed that the hemostatic effect of the sealant in emergency thoracic aortic surgery was reasonably effective. The effective hemostatic rate of emergency cases was 60.2%, whereas the rate in the group of elective cases was 71.6%. Although the difference reached statistical significance, we felt that the hemostatic rate in the emergency group was reasonable, considering the tough background of AAS cases undergoing emergency surgery.

This discussion would not be complete without mentioning the data obtained from the clinical trial of the sealant [11]. The trial had two research arms: (1) elective cases with the sealant and (2) elective cases without the sealant. The hemostasis rate in the second group (elective surgery without the sealant) was 44.4%, which was lower than the rate of 60.2% among emergency cases with the sealant in the present study (Fig. 2). Needless to say, it is not possible to statistically compare the numbers between this study and the clinical trial, as the background of the population is different. Considering, however, that in the clinical trial, (1) patients with aortic dissection were not included, and (2) patients with comorbidities, such as renal dysfunction, a low platelet count, or high fibrin degradation product level, had been excluded, we felt that the hemostatic rate in the emergency case was impressive, as the PMS showed a high hemostatic effect among emergency cases without any significant exclusion criteria.

### The evaluation of the hemostatic effectiveness of the sealant

Although there have been several studies evaluating the hemostatic effect at vascular anastomoses [4, 13–15], there have been few investigating hemostatic agents or hemostatic techniques in acute aortic dissection. Minato et al. [16] showed that rubbing fibrin glue into the anastomoses before reinstituting blood flow resulted in better hemostasis. Those authors measured blood loss by applying gauze sponge around the anastomosis and weighed it to compare the bleeding with that of control patients. Using their method was not feasible, as they started measuring the amount of bleeding immediately after restoring the blood flow through the anastomoses, whereas in our study, the sealant was applied when the surgeons judged that the bleeding was not likely to subside spontaneously. Therefore, there was a certain interval between the blood flow restoration and the timing of applying the sealant, making it impossible to start measuring the amount of bleeding immediately after restoration of blood flow with our protocol. Another approach involves comparing the amount of bleeding during surgery or the amount of blood products transfused during the surgery. However, because of the impaired blood coagulation function in patients with AAS, it is difficult to discriminate bleeding from the anastomosis and that from the surgical incision, including the split sternum. In our clinical trial, we have employed visual inspection of bleeding by requesting that surgeons report bleeding as positive or negative, with a judgment of “negative” made if complete hemostasis was obtained (otherwise, positive). To be more objective, recording bleeding from the anastomosis on video might be a good method, but such an approach was not practical for this PMS, as providing the same equipment and standardizing the video recording was not considered possible.

In the present study, we focused on surgeons’ actions to initiate additional hemostatic procedures. This is based on the assumption that the decision to perform further hemostatic procedure depends on each surgeon’s personal criteria, which should be fairly comparable to the criteria of other surgeons, as surgeons who are capable of performing thoracic aortic surgery should all have competent surgical skills and judgement ability.

### Implication of the mechanism being independent of the blood coagulation function

The mechanism of the sealant suggests that the hemostatic effect of the sealant should work under the condition of an impaired coagulation function. In the present study, the sealant was used before neutralizing heparin in 37.1% of the anastomoses. In this group of patients, no further hemostatic procedure was performed in 63.4% of cases. In cases such as aortic root replacement or for the distal anastomosis of the total arch replacement through mid-sternal incision, applying the sealant after heparin reversal is not possible, as reaching the anastomosis is very difficult. An acceptable success rate of 63.4% for hemostasis under a fully heparinized condition implied that this feature would function as an advantage in cases of challenging surgery of the thoracic aorta.

### Non-absorbable nature of the sealant

Unlike other biological sealants, this sealant is not biodegradable. This has raised some concern that the rate of infection or inflammation might be high. In the clinical trial, the rates of pleural effusion were 35.8% and 22.2% (*p* = 0.308) in the control and sealant groups, respectively [11]. In the current PMS, the rate of pleural effusion was 21.2%, which was comparable to that in the control group of the clinical trial. The rate of mediastinitis in this PMS was 2.6%. The rates of mediastinitis reported in previous studies ranged from < 1% to 6% [17–19]. The chemical structure of the cured sealant is a polyurethane. Polyurethane has been used to craft the diaphragm of extracorporeal ventricular devices because of its stable structure. Although the sealant remains around the anastomosis, we consider its risk of causing infection to be no greater than that of felt strips reinforcing the anastomosis, as the nature of this sealant and that of felt strips with regard to remaining around the anastomosis is similar.

### Other applications of the sealant

The sealant was initially approved for anastomoses of the thoracic aorta. The indication was then extended to all vascular anastomoses. Several studies have reported its use for treating left ventricular rupture [20, 21] and reinforcing the suture line of aortic dissection [22] as well as in combination with other hemostatic agents [23]. Because of its powerful hemostatic effect and elastomeric property, numerous applications of the sealant are to be expected. Shimura et al. used this sealant to close the pseudo-lumen of the dissected proximal aortic root to attach the dissected intima to the aortic wall [24]. The elastomeric property of the sealant enabled the creation of a flexible aortic root wall after closing the pseudo-lumen, which made it easy to handle and sew the vascular prosthesis to the reconstructed aortic root stump (personal communication).

### Limitations of the PMS

Because of the nature of the PMS, some parameters or test results were not available, including the operative time, total ischemic time, amount of transfusion, platelet count, and fibrin degradation product values. We, therefore, could not determine to what extent the coagulation function was impaired in emergency cases and were unable to show that the efficiency of the sealant described in emergency cases had been achieved despite an impaired blood coagulation function. We admit that because of the nature of PMS, both authors have conflict of interest as stated in the COI form, and that the study suffers from the limitation with COI. To avoid any biases to occur, we tried to maintain transparency of the data as much as possible and to keep consistency by looking the data from different point of views. The present study also suffers from limitations due to its retrospective study. In addition, to clarify the advantages of the sealant in emergency cases, a group including patients undergoing emergency surgery without the usage of the sealant should have been created. However, this was not possible because of the nature of the PMS. Nevertheless, we at least can speculate that the sealant was effective in emergency cases and under fully heparinized conditions based on the fact that the hemostatic effectiveness was fairly good.

## Conclusion

Using PMS data regarding Matsudaito, a unique sealant that works independent of the blood coagulation function, we were able to show that the sealant was practically effective in emergency surgery for thoracic aorta. To demonstrate its superiority statistically, a prospective randomized study is warranted.
